# Neuropathic Pain in Patients Undergoing Primary Total Knee Arthroplasty: Preval ence and Risk Factors

**DOI:** 10.1055/s-0046-1820456

**Published:** 2026-06-08

**Authors:** Gustavo Waldolato Silva, Rafael Silva E Castro, Gabriela Vieira Brito, Geovanna Reis

**Affiliations:** 1 Department of Orthopedics, Hospital Universitário Ciências Médicas, Belo Horizonte, MG, Brazil; 2 Faculdade Ciências Médicas de Minas Gerais, Belo Horizonte, MG, Brazil

**Keywords:** arthroplasty, replacement knee, chronic pain, neuralgia, prevalence, risk factors, artroplastia do joelho, dor crônica, fatores de risco, neuralgia, prevalência

## Abstract

**Objective:**

This study aimed to evaluate the prevalence and risk factors of neuropathic pain (NP) in patients undergoing primary total knee arthroplasty (TKA).

**Methods:**

The present prospective observational study included patients aged 18 years or older who were candidates for TKA, conducted between October 2023 and October 2024 at a university hospital. Data were collected from medical records and through the application of a questionnaire assessing socioeconomic variables, comorbidities, prior treatments, and the interview version of the Douleur Neuropathique in 4 questions (DN4i) questionnaire, a validated screening instrument for NP. Paired samples were analyzed using the Wilcoxon, Chi-squared of independence, and Fisher's exact tests, with a significance level set at *p* < 0.05.

**Results:**

Of the 68 participants, 20 (29%) tested positive for NP. Among these, most were female (80%, *p* = 0.015). Pain intensity was significantly higher in this group, with 90% reporting VAS scores between 8 and 10. There were no significant associations with obesity, diabetes, or the use of pain modulators. Quality of life was more adversely affected in patients with NP, who reported poorer general health status and greater limitations in physical and social activities.

**Conclusion:**

There is a high prevalence of NP among patients undergoing TKA, particularly in females, and is associated with worse quality of life and greater pain intensity. Systematic screening using the DN4i questionnaire may improve diagnosis and support the development of individualized therapeutic strategies.

## Resumo

**Objetivo:**

Avaliar a prevalência e os fatores de risco de dor de característica neuropática (DN) em pacientes que serão submetidos a ATJ primária.

**Métodos:**

Trata-se de um estudo prospectivo observacional realizado com pacientes acima de 18 anos, candidatos a ATJ, durante o período de outubro de 2023 a outubro de 2024, em um hospital universitário. A coleta de dados foi realizada baseada no prontuário dos pacientes e na aplicação de um questionário com variáveis socioeconômicas, comorbidades, tratamentos prévios e o DN4i, instrumento validado de triagem para DN. Para avaliar e correlacionar as amostras pareadas foram utilizados os testes de Wilcoxon, Qui-quadrado de independência e exato de Fisher, considerando nível de significância de *p* < 0,05.

**Resultados:**

Dos 68 participantes, 20 (29%) apresentaram resultado positivo para DN. Dentre eles, a maioria era do sexo feminino (80%, *p* = 0,015). A intensidade da dor foi significativamente maior nesse grupo, com 90% relatando EVA entre 8 e 10. Não foram observadas associações significativas com obesidade, diabetes ou uso de moduladores da dor. A qualidade de vida foi mais impactada nos pacientes com DN, que relataram pior estado geral de saúde e maior limitação em atividades físicas e sociais.

**Conclusão:**

A DN é altamente prevalente em pacientes candidatos à ATJ, principalmente do sexo feminino, e está associada a pior qualidade de vida e maior intensidade de dor. A triagem sistemática com o DN4i pode otimizar o diagnóstico e contribuir para estratégias terapêuticas personalizadas.

## Introduction

Neuropathic pain (NP) is a chronic syndrome defined as “pain arising as a direct consequence of a lesion or disease affecting the somatosensory system.” 1 It differs from nociceptive pain by presenting characteristics such as dysesthesia, electric shock-like sensations, and burning pain. 2 In Brazil, its prevalence among patients with chronic pain is estimated at 14.5%. 3

Literature data indicate that female gender, advanced age, and the use of pain modulators are associated with NP. 4 5 6 In patients with diabetes mellitus (DM), adequate glycemic control reduces the risk of developing NP and its impacts. 7

Among the screening tools for NP, the interview version of the Douleur Neuropathique in 4 questions (DN4i) is a simplified version of the DN4, comprising only the interview component, which allows its application in settings such as telephone-based assessments. A score of up to 3 indicates a positive result for NP. 8 The DN4 is considered practical compared with other instruments due to its relatively small number of items and high ability to discriminate neuropathic from nociceptive pain. 9

The global prevalence of gonarthrosis, or knee osteoarthritis (OA), is estimated at 3.8%. This disease causes significant functional limitation, primarily due to chronic pain. OA is more common in women, and the typical peak is around 50 years of age. Factors such as population ageing, increased longevity, obesity, and higher functional demands have contributed to the growing number of primary total knee arthroplasties (TKAs). 10 11

In the United States, the demand for primary TKA is projected to increase by 673% by 2030, reaching 3.48 million procedures annually. Globally, there is an average of 175 TKAs per 100,000 inhabitants. 12

The goal of TKAs to restore function and relieve pain in patients with gonarthrosis. However, in clinical practice, some patients experience incomplete functional recovery and persistent pain, even in the absence of surgical complications. Therefore, this study aimed to investigate the prevalence and risk factors for chronic pain with neuropathic characteristics in patients with gonarthrosis who are candidates for primary TKA.

## Materials and Methods

The present prospective observational study included patients scheduled for primary TKA between October 2023 and October 2024 at a university hospital. The Institutional Research Ethics Committee approved this study under the number CAAE 76569923.9.0000.5125.

The study was based on a convenience sample, as all patients evaluated by orthopedic surgeons at this hospital with an indication for primary TKA received an invitation to participate. Eligibility criteria included age 18-years or older and being clinically fit to undergo the surgery. Patients were excluded if they refused to participate, if telephone contact was unsuccessful, or if they were not undergoing primary TKA (e.g., revision procedures). All subjects included had to sign the written informed consent form before surgery.

Although this study relied on a convenience sample, a previous sample size calculation was performed to estimate prevalence. Assuming an expected prevalence of NP in patients with knee OA of 25%, a maximum absolute error of 10%, and a 95% confidence level, the minimum required sample size was 65 participants. Therefore, the final sample (68 patients) exceeded the estimated minimum.

Two orthopedic surgeons from the clinical staff, who routinely perform knee arthroplasty, participated in the development and administration of a structured questionnaire via telephone. The questionnaire included data from medical records, as well as socioeconomic variables (education level and place of residence), previous treatments (e.g., physical therapy, intra-articular injections), use of pain medications, and comorbidities. The DN4i questionnaire, in a version validated and translated into Brazilian Portuguese, including only the interview component, was chosen for allowing telephone-based administration, which enabled standardized data collection and ensured inclusion of all participants. Postoperative follow-up did not occur at the same location or time. The instrument consists of seven items across two domains: the first assesses pain characteristics (e.g., burning, painful cold, and electric shock sensations), and the second evaluates associated symptoms and dysesthesias (e.g., tingling, pins-and-needles sensations, numbness, and itching). The DN4i score ranges from 0 to 7, with each item scored as “yes” (1) or “no” (0). A total score of up to 3 indicates the presence of NP. 8

The questionnaire also included the visual analog scale (VAS) for pain and the Short Form-8 (SF-8). The first was used to assess pain intensity, with scores ranging from 0 to 2 indicating mild pain, 3 to 7 moderate, and 8 to 10 severe. 13 The second is a shortened version of the Short Form-36 and comprises eight domains designed to evaluate the physical and mental aspects of quality of life over the previous 4 weeks. 14

An independent researcher conducted the statistical analysis. Descriptive statistics were calculated to characterize the sample. Quantitative variables were summarized using measures of central tendency (mean or median), while categorical variables were presented as frequencies and percentages.

The Wilcoxon signed-rank test was used to compare paired nonparametric data, ensuring appropriate statistical analysis according to data type and study conditions. The Chi-squared test of independence was used to assess associations between categorical variables in larger samples, while Fisher's exact test was applied to contingency tables with smaller expected frequencies, ensuring the validity of results in smaller groups. In all statistical tests, the significance level was set at *p* < 0.05, with 95% confidence intervals (CIs). Data analysis was performed using the IBM SPSS Statistics Base (IBM Corp.), version 22.0.

## Results

**Fig. 1:**
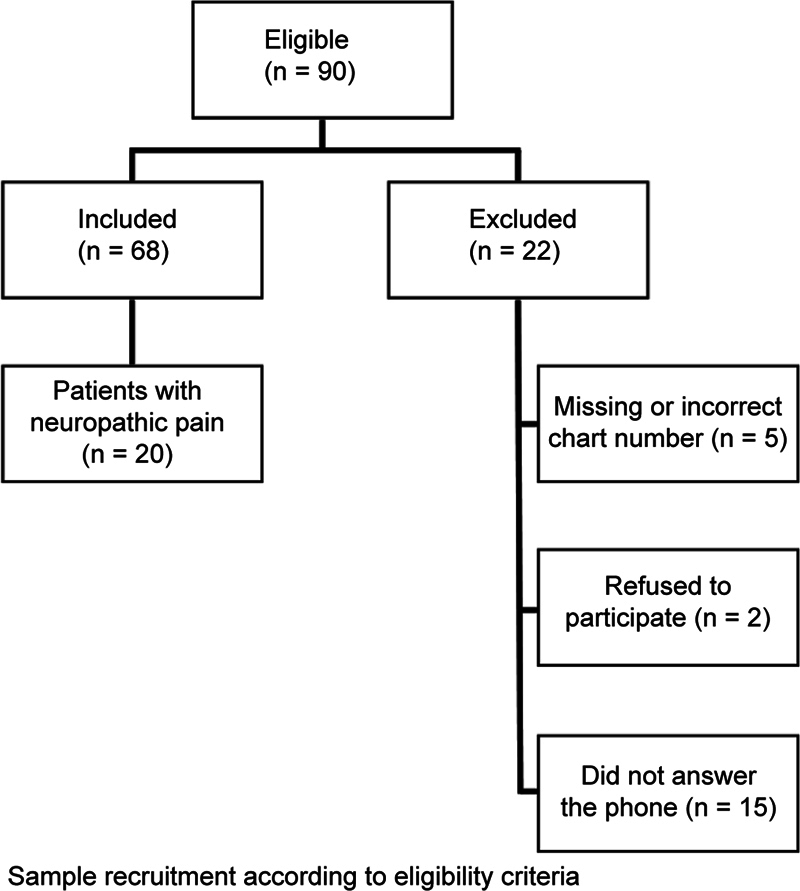
Sample recruitment according to eligibility criteria.

A total of 90 participants were eligible for the study, of whom 68 had complete and analyzable data. Fig. 1 presents the participant flow. The median age was 67 years (interquartile range [IQR]: 63–73), with 59% aged ≥ 65 years, and a predominance of females (57%). Obesity was identified in 41% (n = 28) of the sample. The most frequent comorbidities were systemic arterial hypertension (SAH: 76%) and DM (29%). Regarding pharmacological treatment, 19% (n = 13) of the participants were using pain modulators, while 72% (n = 49) used other types of analgesics. In terms of previous treatments, 59% (n = 40) had undergone physical therapy, and 19% (n = 13) reported intra-articular injections. The DN4i score was positive for NP in 29% (n = 20) of patients. Table 1 presents the socioeconomic and clinical characteristics of participants with analyzable data.

**Table 1:** Socioeconomic and clinical characteristics of the sample

Characteristics	N = 68 ^a^
**Age, years**	67 (63–73)
≥ 65	40 (59%)
< 65	28 (41%)
**Gender**	
Female	39 (57%)
Male	29 (43%)
**Area**	
Belo Horizonte	34 (50%)
State of Minas Gerais	34 (50%)
**Educational level**	
Incomplete	57 (84%)
Higher education	11 (16%)
**BMI**	
Non-obese	40 (59%)
Obese	28 (41%)
**DM**	
No	48 (71%)
Yes	20 (29%)
**SAH**	
No	16 (24%)
Yes	52 (76%)
**Use of pain modulators**	
No	49 (72%)
Not under any medication	6 (8.8%)
Yes	13 (19%)
**Previous treatments**	
Physical therapy	40 (59%)
Infiltration	13 (19%)
None	15 (22%)
**DN4i result**	
Negative	48 (71%)
Positive	20 (29%)

**Abbreviations:** BMI, body mass index; DM, diabetes mellitus; DN4i, Douleur Neuropathique in 4 questions interview version; SAH, systemic arterial hypertension. **Note:** ^*a*^ Values expressed as medians (Q1, Q3); n (%).

Among patients with NP, the prevalence of this condition was higher in females (80%) compared to males (20%; *p* = 0.015). Most patients reported severe pain intensity according to the VAS score (79%), with an even higher proportion among those with NP (90%). In the quality-of-life domain assessed by the SF-8, 10% of patients with NP reported their health status as “very poor,” with no cases in the group without. Additionally, patients with NP showed greater impairment in physical and social activities. Although 41% of participants were obese, there was no significant association between body mass index (BMI) and the presence of NP ( *p* = 0.7).

Regarding comorbidities, there were no statistically significant differences for SAH ( *p* = 0.6) or DM ( *p* ≥ 0.9) between patients with and without NP. Moreover, the use of pain-modulating medications did not differ significantly between groups. Table 2 presents a cross-tabulation of DN4i scores with other variables.

**Table 2:** Cross-tabulation of DN4i scores with other variables

Characteristics	Total N = 68 ^a^	Neuropathic pain		*p* -value ^b^
		No N = 48 ^a^	Yes N = 20 ^a^	
**Age, years**	67 (63–73)	67 (64–73)	67 (63–72)	
≥ 65	40 (59%)	29 (60%)	11 (55%)	0.8
< 65	28 (41%)	19 (40%)	9 (45%)	0.7
**Gender**				**0.015**
Female	39 (57%)	23 (48%)	16 (80%)	
Male	29 (43%)	25 (52%)	4 (20%)	
**Area**				0.3
Belo Horizonte	34 (50%)	22 (46%)	12 (60%)	
State of Minas Gerais	34 (50%)	26 (54%)	8 (40%)	
**Educational level**				> 0.9
Incomplete	57 (84%)	40 (83%)	17 (85%)	
Higher education	11 (16%)	8 (17%)	3 (15%)	
**BMI**				0.7
Non-obese	40 (59%)	29 (60%)	11 (55%)	
Obese	28 (41%)	19 (40%)	9 (45%)	
**DM**				0.6
No	48 (71%)	33 (69%)	15 (75%)	
Yes	20 (29%)	15 (31%)	5 (25%)	
**SAH**				> 0.9
No	16 (24%)	11 (23%)	5 (25%)	
Yes	52 (76%)	37 (77%)	15 (75%)	
**Use of pain modulators**				> 0.9
No	49 (72%)	35 (73%)	14 (70%)	
Not under any medication	6 (8.8%)	4 (8.3%)	2 (10%)	
Yes	13 (19%)	9 (19%)	4 (20%)	
**Previous treatments**				> 0.9
Physical therapy	40 (59%)	28 (58%)	12 (60%)	
Infiltration	13 (19%)	9 (19%)	4 (20%)	
No	15 (22%)	11 (23%)	4 (20%)	
**VAS**				0.2
Intense (8–10)	54 (79%)	36 (75%)	18 (90%)	
Moderate (3–7)	14 (21%)	12 (25%)	2 (10%)	
**Q1-SF8: Overall health status**				0.12
Good	39 (57%)	29 (60%)	10 (50%)	
Very poor	2 (2.9%)	0 (0%)	2 (10%)	
Fair	23 (34%)	17 (35%)	6 (30%)	
Poor	4 (5.9%)	2 (4.2%)	2 (10%)	
**Q2-SF8: Physical activities**				0.7
Inability to do physical activities	3 (4.4%)	2 (4.2%)	1 (5.0%)	
Very little	1 (1.5%)	1 (2.1%)	0 (0%)	
Regular	8 (12%)	7 (15%)	1 (5.0%)	
Significant	56 (82%)	38 (79%)	18 (90%)	
**Q3-SF8: Daily work**				0.11
Inability to work daily	5 (7.4%)	1 (2.1%)	4 (20%)	
Very little	1 (1.5%)	1 (2.1%)	0 (0%)	
None	1 (1.5%)	1 (2.1%)	0 (0%)	
Regular	11 (16%)	8 (17%)	3 (15%)	
Significant	50 (74%)	37 (77%)	13 (65%)	
**Q4-SF8: Body aches in the last 4 weeks**				**0.046**
Mild	17 (25%)	14 (29%)	3 (15%)	
Severe	15 (22%)	8 (17%)	7 (35%)	
Moderate	32 (47%)	25 (52%)	7 (35%)	
Very severe	4 (5.9%)	1 (2.1%)	3 (15%)	
**Q5-SF8: Vitality in the last month**				0.2
High	30 (44%)	24 (50%)	6 (30%)	
Low	11 (16%)	8 (17%)	3 (15%)	
Very high	1 (1.5%)	0 (0%)	1 (5.0%)	
None	3 (4.4%)	1 (2.1%)	2 (10%)	
Regular	23 (34%)	15 (31%)	8 (40%)	
**Q6-SF8: Social activities**				0.3
Inability to engage in social activities	2 (2.9%)	1 (2.1%)	1 (5.0%)	
Very little	3 (4.4%)	1 (2.1%)	2 (10%)	
None	49 (72%)	35 (73%)	14 (70%)	
Regular	5 (7.4%)	5 (10%)	0 (0%)	
Significant	9 (13%)	6 (13%)	3 (15%)	
**Q7-SF8: Emotional issues**				>0.9
Mild	12 (18%)	9 (19%)	3 (15%)	
Moderate	6 (8.8%)	4 (8.3%)	2 (10%)	
None	45 (66%)	31 (65%)	14 (70%)	
Significant	5 (7.4%)	4 (8.3%)	1 (5.0%)	
**Q8-SF8: Limitation of daily activities due to personal or emotional problems**				>0.9
Very little	4 (5.9%)	3 (6.3%)	1 (5.0%)	
None	59 (87%)	42 (88%)	17 (85%)	
Regular	3 (4.4%)	2 (4.2%)	1 (5.0%)	
Significant	2 (2.9%)	1 (2.1%)	1 (5.0%)	

**Abbreviations:** BMI, body mass index; DM, diabetes mellitus; DN4i, Douleur Neuropathique en 4 questions interview version; SAH, systemic arterial hypertension; SF-8, Short Form-8; VAS, visual analog scale. **Notes:** ^*a*^ Median (Q1, Q3); n (%). ^*b*^ Wilcoxon rank-sum test; Chi-squared test of independence; Fisher's exact test.

## Discussion

This study demonstrated that the prevalence of NP in patients scheduled for primary TKA was 29%, with a significant predominance in females (80%, *p* = 0.015). Additionally, patients with NP reported higher pain intensity, with 90% (n = 18) classifying it as severe (VAS: 8–10), and experienced a greater impact on quality of life, with 10% (n = 2) reporting a “very poor” health status, as well as greater physical and social limitations.

A potential explanation for the high prevalence of NP in this group is the presence of known risk factors, such as female gender and older age, which is consistent with previous epidemiological studies on NP. Other investigations also reported an association with the female gender, attributing it to hormonal differences, including the role of estrogens in pain modulation and increased central sensitization, as described by Torrance et al. 4 and Bouhassira et al. 5

However, there were no significant associations between NP and comorbidities such as obesity, SAH, or DM, contrasting with studies that identify these conditions as potential predisposing factors. 6 7 15

The high intensity of NP and its negative impact on quality of life corroborate findings in the literature that associate this condition with greater psychological and functional burden. 7 16 17 This relationship has been observed in studies using questionnaires such as the EuroQol 5-Dimension (EQ-5D) and the 12-Item Short Form Health Survey, version 2 (SF-12v2), which serve the same purpose as the SF-8 and demonstrate worse physical and mental scores in affected patients. 17 The negative impact also includes limitations in daily and social activities, factors commonly observed in orthopedic populations with chronic pain.

Greater social and daily restrictions reinforce the need for multidisciplinary approaches in managing this population. Some authors, such as Helito et al. 17 and Zolio et al., 18 emphasize that early detection NP may guide more effective rehabilitation strategies, including specialized physical therapy and the use of neuromodulatory medications. Recent studies indicate that the prevalence of NP in patients with OA ranges from 20 to 28.6%, with a significant impact on pain intensity and quality of life. 17 In a multicenter study, patients with NP showed greater physical and mental impairment, supporting findings that this condition is associated with greater functional limitation and worse surgical outcomes. 18

The high proportion of patients with severe pain (VAS: 8–10) in the neuropathic group reinforces the greater severity of NP, which has been described as a factor that amplifies functional disability and reduces quality of life. 4 In the context of TKA, these findings highlight the importance of early identification of patients with NP to prevent negative impacts on postoperative outcomes. 19

Although nonpharmacological interventions such as physical therapy were widely used, no significant difference was observed between patients with and without NP, suggesting that additional approaches are required to manage this subtype of pain. Studies suggest that pain-modulating agents, such as anticonvulsants and antidepressants, may be effective in NP management. 4 5 6

This study has some limitations. First, data collection was conducted through telephone interviews using the DN4i. Although this questionnaire facilitates patient access and standardizes instrument administration, it precludes in-person assessment of neurological signs and may increase the risk of recall bias and misinterpretation of questions by participants. Moreover, no specific cognitive screening tools were used during the calls, which may have resulted in less accurate responses by older patients with potential cognitive impairment.

Second, some comorbidities potentially related to NP (such as diabetic peripheral neuropathy, lumbar radiculopathies, or other disorders of the peripheral nervous system) may have influenced DN4i scores and were not assessed in detail, limiting the ability to distinguish NP attributable exclusively to knee OA from other concomitant causes. Lastly, the observational design and the single-center sample limit the generalizability of the findings, preventing the establishment of causal relationships between potential risk factors and NP presence.

The main strengths of this study include its novelty in investigating NP in patients in the preoperative period of primary TKA, as this is a frequently underdiagnosed and, consequently, undertreated condition in routine orthopedic practice, particularly in this cohort. Additionally, the use of diagnostic tools that allow remote application facilitates patient contact and adherence. The DN4i also enables studies comparing clinical outcomes across different regions.

Further studies, particularly longitudinal investigations, are required to better evaluate the prevalence of NP and the key factors associated with worse functional outcomes. Additionally, expanding epidemiological knowledge of this condition may contribute to improved prognosis through more appropriate therapeutic strategies.

## Conclusion

There was a 29% incidence of NP in patients with gonarthrosis who were candidates for primary TKA. It was also more frequent in females. The presence of NP was associated with greater pain intensity and worse quality-of-life scores, highlighting that this condition is a relevant component of preoperative pain.
